# Five-year change of clinical and complications profile of diabetic patients under primary care: a population-based longitudinal study on 127,977 diabetic patients

**DOI:** 10.1186/s13098-015-0072-x

**Published:** 2015-09-17

**Authors:** Colman Siu Cheung Fung, Eric Yuk Fai Wan, Fangfang Jiao, Cindy Lo Kuen Lam

**Affiliations:** Department of Family Medicine and Primary Care, The University of Hong Kong, 3/F Ap Lei Chau Clinic, 161 Main Street, Ap Lei Chau, Hong Kong

**Keywords:** Diabetes mellitus, Primary care, Health service, Chinese, Population-based

## Abstract

**Background:**

Number of diabetic patients under public primary care in Hong Kong rose from 150,157 (2009) to 173,015 (2013). This study aimed to track the 5-year change of their outcomes and care standard after the introduction of quality enhancement programmes.

**Methods:**

Longitudinal study was conducted on a group of diabetic patients who received continuous care under public primary care between 2009 and 2013. Socio-demographic and clinical data was retrieved from central database. The standard of care in terms of proportion of patients achieving haemoglobin A1c (HbA1c), systolic and diastolic blood pressure (SBP and DBP), and low density lipoprotein-cholesterol (LDL-C) target levels, mean parameter changes, and 5-year cumulative incidence of major complications were assessed. Outcomes between 2009 and 2013 were compared by McNemar’s test for proportion of patients treated to targets and paired t-test for continuous outcome parameters.

**Results:**

A group of 127,977 diabetic patients who had continuous follow-up between 2009 and 2013 were assessed. A significantly higher proportions of patients achieving targets of HbA1c (<7 %), SBP (<130 mmHg), DBP (<80 mmHg), LDL-C (<2.6 mmol/L), triglyceride (<1.7 mmol/L), and high density lipoprotein-cholesterol (>1.0 or 1.3 mmol/L) were observed (p < 0.001). There was a significant drop in the mean values of HbA1c (7.2–7.0 %), SBP (136.9–131.3 mmHg), DBP (75.4–72.1 mmHg), LDL-C (3.1–2.4 mmol/L), triglyceride (1.7–1.4 mmol/L), and body mass index (25.6–25.3 kg/m^2^). More patients (0.6 % raised to 3.5 %) used insulin in addition to their oral anti-diabetic drugs for their management, and a significant boost (from 9.0 to 55.0 %) was on statin use. 5-year cumulative incidence of any major diabetic complication was 6.2 %.

**Conclusions:**

Standard of public primary care for diabetic patients enhanced from 2009 to 2013, as reflected by the improvement in outcomes of care. It could be related to the implementation of the territory-wide quality enhancement programmes in all public primary care clinics since 2009, with coverage increasing from 3.1 % (2009) to 81.9 % (2013).

Clinical trial number and registry: NCT02034695, ClinicalTrials.gov

**Electronic supplementary material:**

The online version of this article (doi:10.1186/s13098-015-0072-x) contains supplementary material, which is available to authorized users.

## Background

Diabetes mellitus (DM) is a significant major chronic disease and it was estimated 1 in 12 people was affected by diabetes in the world [1]. The World Health Organization projected that by 2030, diabetes would be the seventh most common cause of death in the world [2]. A good control of DM is crucial because DM is closely linked to various complications, ranging from different cardiovascular diseases (including myocardial infarction, cerebrovascular diseases, etc.) to microvascular diseases like diabetic retinopathy [including sight-threatening diabetic retinopathy (STDR)] and diabetic nephropathy [including end stage renal failure (ESRF)]. Health spending on diabetes increased to 548 billion, accounting for 10.8 % of total health expenditure worldwide in 2013 [1]. Patients with DM complications significantly increased medical costs compared with patients without DM complications [3]. A huge number of diabetic patients and substantial public health burden will be further exacerbated by the rapidly ageing population in the world. To prevent diabetic complications, many overseas national professional associations have developed guidelines on the management of diabetes [4–7]. Similar initiative was also called for to assure the management and quality of care of diabetes for the population in Hong Kong (HK).

Hong Kong, being an international city with many people leading a Western lifestyle, hosted a significant and increasing number of diabetic patients in recent years. Local data showed that the prevalence of diabetic mellitus was 9.92 % (diagnosed plus undiagnosed) [1]. Total annual costs for diagnosed Type 2 DM patients in HK were estimated to take up 6.4 % of the total expenditure of the public healthcare sector in 2004 [8]. In order to enhance the quality of care of patients with chronic diseases like diabetes mellitus under the public primary care setting, various territory-wide quality enhancement programmes have been introduced in phases, including Risk Assessment and Management Programme-Diabetes Mellitus (RAMP-DM), Patient Empowerment Programme, Nurse Allied Health Clinic, Call Centre, etc. RAMP-DM is exclusively for diabetic patients, and it has started implementation since 2009, with its coverage raised from 3.1 % in 2009 to 81.9 % in 2013 of all the diabetic patients under the care of public primary healthcare sector. The details of the RAMP-DM had been illustrated in our previous protocol paper [9]. Diabetic patients are encouraged to live and work in the community, and primary care approach of community-based care are appropriate to majority of the diabetic patients who do not require specialist care [10, 11]. In spite of this predominant chronic disease in the society, there were no formal standardized guidelines or recommendations provided to the local health care doctors as a local reference on how their diabetic patients should be managed before 2010. In 2010, the HK Reference Framework for Diabetes Care for Adults in Primary Care Setting was published and served as a population-wide guideline for strengthening community-based primary care, focusing on prevention, and quality improvement in the management of DM [12]. Previous studies demonstrated that healthcare systems relying more on primary care rather than specialist care produce better population health outcomes, enhance continuity and access to health care [13–17]. Nevertheless, currently, limited primary care and population-based data on patient’s clinical and complications profile of diabetic patients was available to identify the needs of the practices and population. Continued territory-wide evaluation of diabetes control in the population is vital to sustain improvement in diabetic care and inform health policy makers in service planning and resource allocation to prevent the development of complications and preserve quality of life of the diabetic population.

The aims of this study were to track the recent 5-year changing diabetic care by reviewing the clinical and complication profile, to evaluate the trend in risk-factor control, and to identify the needs and gaps of the practices among diabetic patients based on the 5-year population-based data from 2009 to 2013.

## Methods

### Study design

A territory-wide longitudinal study was conducted on a group of diabetic patients who received continuous care under public primary care between 2009 and 2013. Patients with a clinical diagnosis of Type 1 or Type 2 DM, and receiving primary care in general outpatient clinics of Hospital Authority (HA) between 1 January 2009 and 31 December 2009 were included in the study. DM was identified with the International Classification of Primary Care-2 (ICPC-2) code of ‘T89’ or ‘T90’ through the Clinical Management System (CMS) database of HA. Diabetic patients who did not have records of follow-up between 1 January 2013 and 31 December 2013 were excluded for analysis. Relevant socio-demographic and clinical data was retrieved from central CMS of HA database. Due to an enormous subsidized public healthcare policy in HK, the majority of diabetic patients are managed in public outpatient clinics or hospitals governed by the HA, which provides 90 % of in-patients service in HK [18, 19].

Ethics approval of this study was granted by the Institutional Review Board of the University of Hong Kong/Hospital Authority Hong Kong West (UW 10-369), Hong Kong East (HKEC-2010-093), Kowloon East and Kowloon Central (KC/KE-10-0210/ER-3), Kowloon West (KW/EX/10-317 (34-04)), New Territories East (CRE-2010.543), and New Territories West clusters (NTWC/CREC/1091/12) and clinical trial registry (NCT02034695, ClinicalTrials.gov).

### Social-demographics and clinical profile

The medical records of hospital admissions and outpatient clinic attendance in all public hospitals and outpatient clinics were retrieved from the administrative database of HA. Socio-demographics of patients included sex, age, smoking status, alcohol drinking habit and duration of diabetes. Clinical variables included body mass index (BMI), waist–hip ratio, hemoglobin A1c (HbA1c), systolic and diastolic blood pressure (SBP and DBP), lipid profile [low density lipoprotein-cholesterol (LDL-C), high density lipoprotein-cholesterol (HDL-C), total cholesterol (TC), TC/HDL-C ratio and triglyceride] and urine albumin-to-creatinine ratio (ACR). Treatment modalities included the use of antidiabetic drugs (e.g. metformin, sulphonylurea, gliptin and insulin, etc.), anti-hypertensive drugs [e.g. angiotensin converting enzyme inhibitor (ACEI) or angiotensin receptor blocker (ARB), β-blocker, calcium channel blocker (CCB), diuretic], lipid-lowering agents (Statin and fibrate) and aspirin. The latest available record before and closest to 31 December 2009 and 31 December 2013 for each patient was used to represent the data of 2009 and 2013 respectively.

### Diabetic complications identification and definition

The medical records documented the diagnosis coding system of ICPC-2 and International Classification of Diseases, Ninth Edition, Clinical Modification (ICD-9-CM) for each outpatient visit and hospital admission respectively. In this study, Coronary Heart Disease (CHD) (ischaemic heart disease, myocardial infarction, coronary death or sudden death) was defined as earliest date of diagnosis with either ICPC-2 of K74 to K76 or ICD-9-CM of 410.x, 411.x to 414.x, 798.x. Stroke (fatal and non-fatal stroke) was defined as earliest date of diagnosis with either ICPC-2 of K89 to K91 or ICD-9-CM of 430.x to 438.x. Heart failure was defined as earliest date of diagnosis with either ICPC-2 of K77 or ICD-9-CM of 428.x. Cardiovascular disease (CVD) is defined as the presence of any of CHD, heart failure and stroke ICPC-2 or ICD-9-CM codes as above. STDR (proliferative diabetic retinopathy, retinal haemorrhage, maculopathy and blindness) was defined as earliest date of diagnosis with ICD-9-CM of 362.02, 362.07, 362.31 362.81 and 369.x and ESRF was defined as earliest date of diagnosis with ICD-9-CM of 250.3x, 585.x, 586.x.

### Measure outcomes

Primary outcome measures were the change in proportions of patients achieving treatment targets, namely, HbA1c <7 %, SBP <130 mmHg, DBP <80 mmHg, LDL-C <2.6 mmol/L, TG <1.7 mmol/L, and HDL-C >1 mmol/L (male) or >1.3 mmol/L (female), etc. Secondary outcome measures were the changes in these clinical variables, drug use pattern, and the 5-year cumulative incidence of major DM complications.

### Data analysis

Descriptive statistics were used to show patient’s socio-demographics, clinical variables, and drug usage pattern. Outcomes between 2009 and 2013 were compared by McNemar’s test for proportion of patients treated to targets and paired t-test for mean level of continuous outcome parameters. For the 5-year cumulative incidence of major DM complications, patients without corresponding DM complications at baseline were included into the calculation.

## Results

**Fig. 1:**
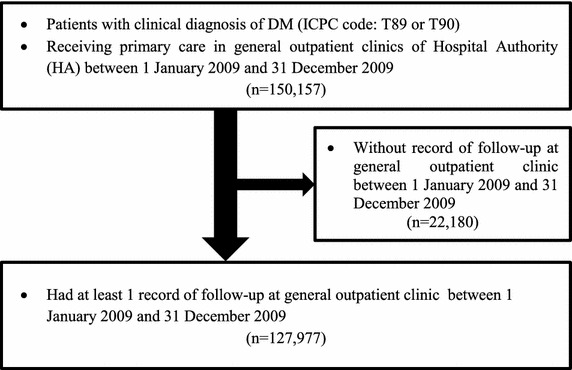
Flow chart of subjects. The flow chart showed the participant enrollment in each stage. *DM* diabetes melltius

The flow of diabetic subjects in the study was summarized in Fig. 1. A total of 150,157 diabetic patients received public primary care at 2009. Among these patients, 127,977 (85.2 %) subjects were found continuously receiving public primary care at 2013 and their data was included for analysis in this study.

The data completion rates for socio-demographic and clinical parameters in 2009 and 2013 were shown in Additional file 1. Clinical data (e.g., BMI, waist–hip ratio, HbA1c, SBP, DBP, full lipid profile and urine ACR, etc.,) completion rate improved significantly. Table 1 and Additional file 2 showed the socio-demographics of the 127,977 DM patients in 2009. Mean age was 64.0. Female was around 10 % more than male.

**Table 1:** Socio-demographic of the 127,977 diabetic subjects at 2009

	Subjects (N = 127,977)
Socio-demographic, (mean ± SD)	
Age, year	64.0 ± 11.2
<65	52.5 %
≥65	47.5 %
Sex	
Female	55.8 %
Male	44.2 %
Smoking status	
Non-smoker	77.7 %
Current smoker	5.6 %
Ex-smoker	16.7 %
Drinking status	
Non-drinker	76.4 %
Current drinker	2.8 %
Social drinker	12.9 %
Ex-drinker	7.8 %
Education level	
No formal education/primary	20.1 %
Primary	39.6 %
Secondary/tertiary	35.6 %
Tertiary	4.7 %
Duration of DM, year	7.1 ± 6.0
≤5 years	41.7 %
5–10 years	30.0 %
>10 years	28.3 %

*DM* diabetes mellitus, *BMI* body mass index, *HbA1c* haemoglobin A1c, *SBP* systolic blood pressure, *DBP* diastolic blood pressure, *LDL-C* low-density lipoprotein-cholesterol, *TC* total cholesterol, *HDL-C* high-density lipoprotein-cholesterol, *ACR* albumin/creatinine ratio

**Fig. 2:**
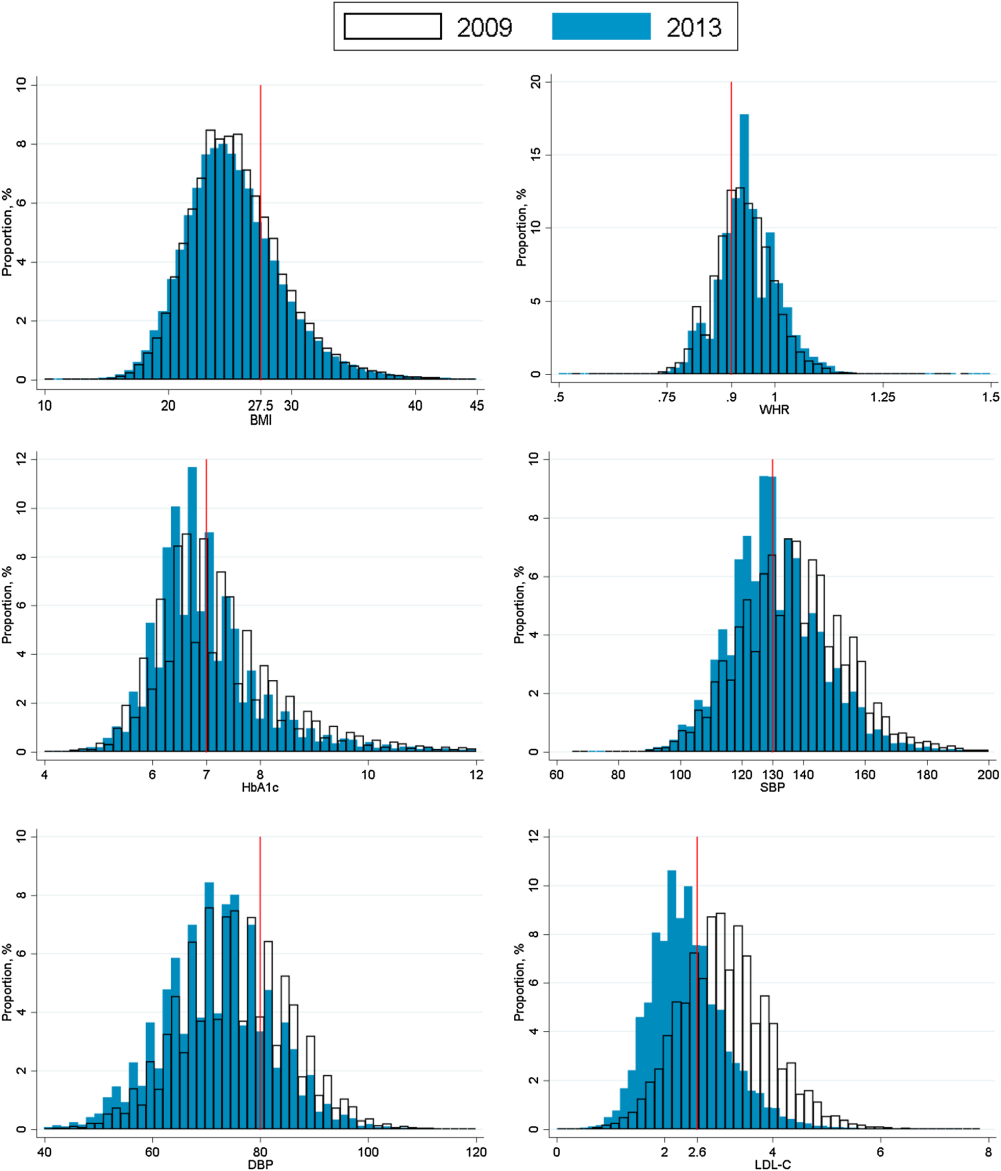
Distribution of clinical variables in 127,977 diabetic subjects between 2009 and 2013. The distribution of clinical variables including BMI, WHR, HbA1c, SBP, DBP, LDL-C among 127,977 diabetic subjects were denominated at 2009 (*white area* with *black borders*) and 2013 (*blue area*). The *red line* indicates the target standard of the clinical variables. *BMI* body mass index, *WHR* waist–hip ratio, *HbA1c* haemoglobin A1c, *SBP* systolic blood pressure, *DBP* diastolic blood pressure, *LDL-C* low-density lipoprotein-cholesterol

**Fig. 3:**
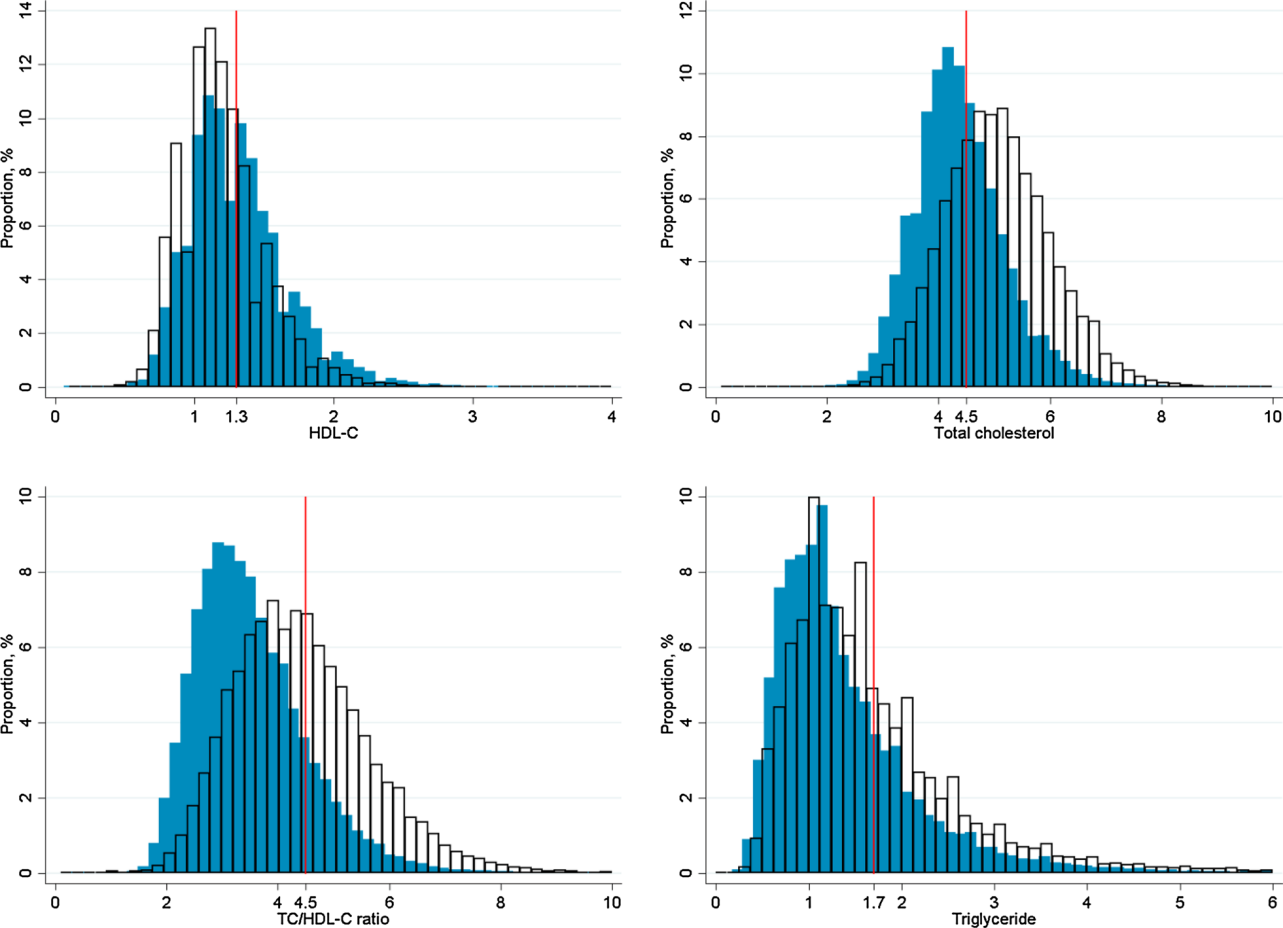
Distribution of clinical variables in 127,977 diabetic subjects between 2009 and 2013. The distribution of clinical variables including HDL-C, TC, TC/HDL-C ratio and Triglyceride among 127,977 diabetic subjects were denominated at 2009 (*white area* with *black borders*) and 2013 (*blue area*). The *red line* indicates the target standard of the clinical variables. *HDL-C* high-density lipoprotein-cholesterol, *TC* total cholesterol

Tables 2, 3 and Figs. 2, 3 compared their clinical variables at 2009 and 2013. A significant increase of proportions of patients achieving treatment targets, including HbA1c <7 % (increased from 47.5 to 56.5 %), SBP <130 mmHg (increased from 47.5 to 56.5 %), DBP <80 mmHg (increased from 65.7 to 77.5 %), LDL-C <2.6 mmol/L (increased from 25.9 to 65.6 %), TG <1.7 mmol/L (increased from 61.6 to 74.9 %), and HDL-C >1.0 mmol/L (male) or >1.3 mmol/L (female) (increased from 47.0 to 60.6 %) were observed (p < 0.001). Mean values of clinical variables also dropped significantly including HbA1c (from 7.2 to 7.0 %), SBP (from 136.9 to 131.3 mmHg), DBP (from 75.4 to 72.1 mmHg), LDL-C (from 3.1 to 2.4 mmol/L), TG (from 1.7 to 1.4 mmol/L), and BMI (from 25.6 to 25.3 kg/m^2^). On the other hand, more proportion of diabetic patients had central obesity (raised from 77.1 to 81.3 %) and increased ACR (raised from 23.0 to 26.3 %).

**Table 2:** Change in levels of clinical variables among 127,977 diabetic subjects between 2009 and 2013

	2009	2013	Paired difference	P-value
Clinical parameters (mean ± SD)				
BMI, kg/m^2^	25.6 ± 3.9	25.3 ± 3.9	−0.3 ± 1.8 (n = 71,224)	<0.001*
Waist–hip ratio	0.9 ± 0.2	0.9 ± 0.1	0.01 ± 0.2 (n = 47,714)	<0.001*
HbA1c, %	7.2 ± 1.2	7.0 ± 1.1	−0.2 ± 1.3 (n = 113,066)	<0.001*
Systolic blood pressure, mmHg	136.9 ± 16.9	131.3 ± 15.0	−5.6 ± 19.8 (n = 126,863)	<0.001*
Diastolic blood pressure, mmHg	75.4 ± 10.2	72.1 ± 10.0	−3.3 ± 11.0 (n = 126,863)	<0.001*
LDL-C, mmol/L	3.1 ± 0.8	2.4 ± 0.7	−0.7 ± 0.9 (n = 72,040)	<0.001*
Triglyceride, mmol/L	1.7 ± 1.1	1.4 ± 0.8	−0.3 ± 1.0 (n = 73,631)	<0.001*
Total cholesterol, mmol/L	5.1 ± 0.9	4.3 ± 0.8	−0.7 ± 1.0 (n = 74,574)	<0.001*
HDL-C, mmol/L	1.2 ± 0.3	1.3 ± 0.4	0.1 ± 0.3 (n = 73,406)	<0.001*
TC/HDL-C ratio	4.4 ± 1.3	3.5 ± 1.0	−0.9 ± 1.2 (n = 73,294)	<0.001*
Urine ACR, mg/mmol	4.7 ± 18.6	7.9 ± 35.1	3.2 ± 33.1 (n = 28,250)	<0.001*

*DM* diabetes mellitus, *BMI* body mass index, *HbA1c* haemoglobin A1c, *SBP* systolic blood pressure, *DBP* diastolic blood pressure, *LDL-C* low-density lipoprotein-cholesterol, *TC* total cholesterol, *HDL-C* high-density lipoprotein-cholesterol, *ACR* albumin/creatinine ratio

* Significant difference (P < 0.05) by paired t-test

**Table 3:** Change in proportions of patients achieving treatment targets among 127,977 diabetic subjects between 2009 and 2013

	2009 (%)	2013 (%)	P-value
Clinical parameters (mean ± SD)			
BMI, kg/m^2^			<0.001*
<23 kg/m^2^	25.3	28.5	
≥23 and <27.5 kg/m^2^	47.8	46.5	
≥27.5 and <30 kg/m^2^	15.2	14.0	
≥30 kg/m^2^	11.8	11.0	
Waist hip ratio			<0.001*
≤0.9 male; ≤0.85 female	22.9	18.7	
>0.9 male; >0.85 female	77.1	81.3	
HbA1c, %			<0.001*
<7 %	47.5	56.5	
≥7 %	52.5	43.5	
Systolic blood pressure, mmHg			<0.001*
<130 mmHg	47.5	56.5	
≥130 mmHg	52.5	43.5	
Diastolic blood pressure, mmHg			<0.001*
<80 mmHg	65.7	77.5	
≥80 mmHg	34.3	22.5	
LDL-C, mmol/L			<0.001*
<2.6 mmol/L	25.9	65.6	
≥2.6 mmol/L	74.1	34.4	
Triglyceride, mmol/L			<0.001*
<1.7 mmol/L	61.6	74.9	
≥1.7 mmol/L	38.4	25.1	
Total cholesterol, mmol/L			<0.001*
<4.5 mmol/L	26.4	61.0	
≥4.5 mmol/L	73.6	39.0	
HDL-C, mmol/L			<0.001*
≤1.0 mmol/L male; ≤1.3 mmol/L female	53.0	39.4	
>1.0 mmol/L male; >1.3 mmol/L female	47.0	60.6	
TC/HDL-C ratio			<0.001*
<4.5	55.7	85.4	
≥4.5	44.3	14.6	
Urine ACR, mg/mmol			<0.001*
≤2.5 mg/mmol man; ≤3.5 mg/mmol female	77.0	73.7	
>2.5 mg/mmol man; >3.5 mg/mmol female	23.0	26.3	

*DM* diabetes mellitus, *BMI* body mass index, *HbA1c* haemoglobin A1c, *SBP* systolic blood pressure, *DBP* diastolic blood pressure, *LDL-C* low-density lipoprotein-cholesterol, *TC* total cholesterol, *HDL-C* high-density lipoprotein-cholesterol, *ACR* albumin/creatinine ratio

* Significant difference (P < 0.05) by McNemar’s test

Table 4 showed the change of drug use pattern between 2009 and 2013. More patients were prescribed oral anti-diabetic medications, with metformin being the most commonly prescribed drug (raised from 71.1 to 80.4 %). A lower proportion of patients used sulphonylurea group of drugs and 1.2 % of patients started using gliptin. More patients (raised from 0.6 to 3.5 %) used insulin in addition to their oral anti-diabetic drugs for their management, and a significant boost (from 9.0 to 55.0 %) on statin use was observed.

**Table 4:** Comparison of the drug use pattern of the 127,977 diabetic subjects between 2009 and 2013

Type of therapy	2009 (%)	2013 (%)
Lifestyle modification only	15.7	10.2
Oral anti-diabetic drug only	83.7	86.3
Insulin only	0.2	0.5
Oral anti-diabetic drug + insulin	0.5	3.0
Anti-diabetic drugs		
Metformin	71.1	80.4
Sulphonylurea	57.0	60.4
Gliptin	0.0	1.2
Insulin	0.6	3.5
Others	0.1	0.1
Anti-hypertensive drugs^a^		
None	2.5	3.6
ACEI or ARB	59.4	58.3
β-blocker	39.2	35.5
CCB	73.9	71.0
Diuretic	11.8	10.7
Other anti-hypertensive drugs	15.1	13.8
Lipid lowering drugs		
Statin	9.0 (15.8 %)^b^	55.0 (56.9 %)^b^
Fibrate	4.6	2.5
Aspirin	8.8 (75.8 %)^c^	14.0 (75.7 %)^c^

Others included Acarbose, Glucagon-like peptide-1 agonist, Meglitinides

*ACEI* angiotensin converting enzyme inhibitor, *ARB* angiotensin receptor blocker, *CCB* calcium channel blocker

^a^The denominator was diabetic patients with hypertension (93,234 and 106,361 at 2009 and 2013, respectively)

^b^The bracket shows the proportion of diabetic patients with hyperlipidemia who are put on statin

^c^The bracket shows the proportion of diabetic patients with confirmed CVD who were put on aspirin

Table 5 illustrated the 5-year cumulative incidence of major DM complications in the period from 2009 to 2013. A total of 8,625, 572 and 155 patients with CVD, STDR, and ESRD, respectively, at baseline were excluded for the calculation of 5-year cumulative incidences of corresponding events. Hence, the 5-year cumulative incidences for CVD, STDR, and ESRD were 5.1, 0.7, and 0.8 %, respectively.

**Table 5:** 5-year cumulative incidence of major DM complications among the 127,977 diabetic subjects between 2009 and 2013

Complications	5-year cumulative incidence^a^
Any major complication(s)	6.2 % (7406/118,702)
CVD	5.1 % (6117/119,352)
CHD	2.2 % (2694/124,535)
Heart failure	2.5 % (3043/122,980)
Stroke	1.2 % (1574/122,980)
STDR	0.7 % (934/127,405)
ESRD	0.8 % (1015/127,822)

*CVD* cardiovascular disease, *CHD* coronary heart disease, *STDR* sight-threatening diabetic retinopathy, *ESRD* end stage renal disease

^a^Patients with corresponding complication at baseline were excluded in the calculation of the incidence

## Discussion

This article is by far the latest and largest territory-wide review of the status and control of health parameters of diabetic patients, with a follow-up of a 5-year period. From 2009 to 2013, the quality of care had been enhanced both in terms of the structure, like documentation of the patients’ clinical data, as well as the process, like the annual checking of diabetic patients’ clinical variables. This undoubtedly raised the standard of care being delivered to patients with chronic diseases like diabetes mellitus as previous records were made accessible and thus continuity of care was facilitated. Having all these clinical variables checked and documented allows regular systematic audit of how these patients progress further on.

The standard of care for diabetic patients under primary care in HK, as reflected by the control of HbA1c, BP and LDL-C, is comparable with that achieved in the developed countries such as UK and US [20, 21]. Improvement in coverage of annual checking of key clinical variables may attribute to the more regular monitoring, which allowed early intervention, such as medication intervention. This as a result led to the improvement in the proportions of reaching treatment targets.

All these changes were paralleled with the implement of the territory-wide quality enhancement programmes since 2009. Amongst these programmes, the RAMP-DM coverage rate raised from 3.1 % (2009) to 81.9 % (2013) of all the diabetic patients under public primary care. The details of the RAMP-DM had been illustrated in our previous protocol paper [9]. One of the key impacts was the advocating use of insulin and statin for the better control of diabetes mellitus and the associated hyperlipidemia at the primary care level. The significant increase of patients using insulin in addition to oral anti-diabetic drug, together with the increased proportion of patients using metformin and newer class of oral anti-diabetic drugs like gliptin class are closely linked to the reduction of mean HbA1c from 7.2 to 7.0 % in 5 years, and the increased proportion of patients having HbA1c <7 %. Based on the UK prospective diabetes study [22], every 1 % reduction in mean HbA1c was associated with reductions in relative risk of 14 % for all-cause mortality and 9.9 % for CHD. Therefore the 0.2 % reduction of HbA1c shown in our study has the potential impact of having a reduction of relative risk of 2.8 % for all-cause mortality and 2.0 % for CHD.

Most Chinese patients refused insulin injection as they have the misconception that insulin injection was painful and they had needle phobia, especially in those who have never started insulin [23]. Education on insulin usage helps patients clear these kinds of misconception and allows them to accept the daily injection of insulin and improve glucose control [24]. Coupling the drop in mean HbA1c was the drop in proportion of diabetic patients solely on diet or lifestyle modification alone. This was in-line to the argument that metformin should be initiated early in the management of diabetic patients. Metformin is the first-line oral anti-diabetic drug and recently there were some studies showing its benefit on reducing cardiovascular complications, in addition to that of lowering HbA1c [25, 26]. With the increased proportion of our patients using metformin, we will study the link between metformin use in Chinese patients and cardiovascular risk. Newer classes of oral anti-diabetic drugs like Dipeptidyl Peptidase-4 Inhibitors class were relatively expensive and patients need to self-purchase these newer drugs in most of the circumstances, which lowered the popularity of such drug usage. As aging population and the associated pancreatic function deterioration is foreseen, the effectiveness of these newer class of anti-diabetic drugs has to be studied. The dramatic boost of statin use explained the drop of mean LDL-C from 3.1 to 2.4 mmol/L and the increased proportion of patients achieving LDL-C target of <2.6 mmol/L. A previous study showed that an increment of 1 mmol/L in LDL-C concentration correlates with a 1.57 increased relative risk of coronary heart disease [27], which equated to a 36 % relative risk of CHD for a decrement of 1 mmol/L in LDL-C. Therefore the 0.7 mmol/L reduction of LDL-C shown in our study has the potential impact of having a reduction of relative risk of 25.2 % for CHD. Proportions of diabetic patients with hyperlipidemia being prescribed statin raised tremendously from 15.8 % in 2009 to 56.9 % in 2013. However there was still around one-third (37.5 %) of patients not achieving target LDL-C with around 40 % of diabetic patients with hyperlipidemia still not being given statin in 2013. The underlying reasons needed to be further studied. On the contrary, the number of patients using fibrate was reduced but the overall control of triglyceride improved with dropping of mean TG from 1.7 to 1.4 mmol/L, and more patients achieving target of TG <1.7 mmol/L. One of the hypotheses may be the partial triglyceride lowering effect of statin which was prescribed extensively [28, 29], or the associated reduction in BMI [30, 31].

Both mean SBP and DBP showed significant drop of 5 and 3 mmHg, respectively, and more patients achieving target SBP of <130 mmHg and DBP of <80 mmHg. Previous literature demonstrated that each 10 mmHg decrease in mean SBP was associated with a relative risk of 12 % for all-cause mortality and 13 % for CHD [32]. Hence, the 5 mmHg reduction of SBP shown in our study has the potential impact of having a reduction of relative risk of 6.0 % for all-cause mortality and 6.5 % for CHD. Similarly, this may be explained by the increased use of anti-hypertensive medications within these 5 years. It was noteworthy that calcium channel blocker was the most prevalent anti-hypertensive drugs used in our diabetic patients (over 70 %), while ACEI or ARB ranked only the second (just above 50 %). ACEI or ARB, due to its renal protective effect, are supposed to be beneficial for diabetic patients and are preferred. Proportions of diabetic patients using ACEI or ARB were static at around 59.4–58.3 % between 2009 and 2013. The under use of ACEI or ARB was also reflected by the elevation of mean urine ACR from 4.7 mg/mmol in 2009 to 7.9 mg/mmol in 2013, and the associated increased proportion of patients having urine ACR >2.5 mg/mmol (male) and >3.5 mg/mmol (female). Chinese patients had about 50 % chance of suffering dry cough on ACEI [33, 34]. The alternative use of ARB could help solve the problem while offering renal protective effect [35, 36], despite the cost of ARB over ACEI is an issue. Appropriate prescription of ARB in diabetic patients should be advocated.

In spite of the improvement in the overall mean HbA1c and full lipid profile, the mean BMI dropped only 0.3 kg/m^2^ after 5 years, and the proportion of patients having central obesity raised from 77.1 to 81.3 %. This suggested that body weight and body fitness management may not be feasible if exercise was not taken into account. The impact of exercise should be evaluated on the control of diabetes mellitus, SBP, DBP, and full lipid profile, and should be integrated to the management plan of diabetic patients [37, 38]. Similarly, despite the significant improvement in HbA1c, SBP, DBP, and full lipid profile, the 5-year cumulative incidence of major DM complications including various CVD were shown higher than that of the general population in Asia [39]. Previous studies also showed that diabetic patients were about 2–4 times more likely to have CVD than non-diabetic patients [40, 41]. This difference was expected because hypertension, hyperlipidemia, hyperglycaemic and obesity, those contribute to the risk for developing CVD, occur more frequently in patient with T2DM compared to general population [42]. In this study, there were a total of 6117 new CVD events amongst those without CVD events at baseline in 2009 after 5 years. This may be related to the concurrent elevation of urine microalbumin level, which was shown to be a significant prognostic factor for various DM complications [43]. Use of aspirin was raised from 8.8 % in 2009 to 14.0 % in 2013, and the majority of those patients having complications were put on aspirin. Aspirin usage as a primary preventive measure in diabetic patients was unknown in our locality and worth exploration. Also, patient’s self-management and adherence of therapy play essential role in achieving the goals of diabetes care and thus further studies is needed to identify and evaluate such characteristics of patients.

### Strengths and limitations

The strengths of this study included the territory-wide coverage and tracking for 5 years of all diabetic patients under the care of public primary care across the whole territory of HK. The huge number of diabetic patients being followed up and information being collected could comprehensively reflect the quality of care delivered by the public primary health care system in HK. Besides, the follow-up period of 5 year allows revealing of subtle or slow progress of parameters like BMI, and complications of low incidence rate like ESRD, etc.

There were some limitations in this study. Firstly, only medical records from the public hospitals and clinics were being able to access to capture information of the diabetic patients and the DM-related complications. The patients and events that were under specialist care or in the private sector were not identified. Nevertheless, based on the enormous subsidized public health care policy in HK, there was a substantial discrepancy in medical fees between public and private services, and many of the patients received public healthcare if they developed complications. Our findings were generated from a large scale of population-based database of the public service that provide care to the majority of patients with chronic diseases like diabetes, and a significant proportion of hospital admissions for management of DM-related complications had been covered. Secondly, since data of some of the other quality enhancement programmes are not comprehensive, comparison of individual programmes was not feasible at this moment. Lastly, approximately 95 % of HK population is of Chinese descent and thus our results may not be generalizable to other ethnicities in Asia.

## Conclusions

Our study showed the significant improvements in the management of DM under primary care in HK. Improvement in coverage of annual checking of key clinical variables and patients’ achievement to target values may be attributed by the regular monitoring and early intervention, including use of appropriate medications and the risk-stratification based multidisciplinary DM management (RAMP-DM). There is still room for further enhancement in the diabetes care, including body weight management and counseling in addition to the achievement of optimal targets. Regular evaluation of diabetes control is crucial to identify and sustain improvements in management and care of diabetic patients.

## Additional file

**Additional file 1:** Data completion rate for the 127,977 diabetic patients at 2009 and 2013
**Additional file 2:** Socio-demographic and clinical variables of the 22,180 excluded subjects at 2009

## Abbreviations

DM: diabetes mellitus
STDR: sight threatening diabetic retinopathy
ESRF: end stage renal failure
HK: Hong Kong
CMS: Clinical Management System
BMI: body mass index
HbA1c: hemoglobin A1c
SBP: systolic blood pressure
DBP: diastolic blood pressure
LDL-C: low density lipoprotein-cholesterol
HDL-C: high density lipoprotein-cholesterol
TC: total cholesterol
ACR: albumin-to-creatinine ratio
ACEI: angiotensin converting enzyme inhibitor
ARB: angiotensin receptor blocker
CCB: calcium channel blocker
CHD: coronary heart disease
CVD: cardiovascular disease
RAMP-DM: Risk Assessment and Management Programme-Diabetes Mellitus
