# Supplementary material for: Five-year change of clinical and complications profile of diabetic patients under primary care: a population-based longitudinal study on 127,977 diabetic patients
Source: Diabetol Metab Syndr. 2015 Sep 17;7:79. doi: 10.1186/s13098-015-0072-x (PMC4574224; doi:10.1186/s13098-015-0072-x)
Supplement: Supplementary file 2 — Additional file 2: Socio-demographic and clinical variables of the 22,180 excluded subjects at 2009 [file 13098_2015_72_MOESM2_ESM.docx]

Additional file 2. Socio-demographic and clinical variables of the 22,180 excluded subjects at 2009

|  | Excluded Subjects (N=22,180) |  | Excluded Subjects (N=22,180) |
| --- | --- | --- | --- |
| **Socio-demographic, (mean±SD)** |  | **clinical variables (mean±SD)** |  |
| Age, year | 70.1±13.8 | HbA1c, % | 7.4±1.5 |
| <65 | 33.4% | <7% | 47.9% |
| ≥65 | 66.6% | ≥7% | 52.1% |
| Sex |  | Systolic blood pressure, mmHg | 137.7±19.3 |
| Female | 51.9% | <130mmHg | 33.9% |
| Male | 48.1% | ≥130mmHg | 66.1% |
| Smoking status |  | Diastolic blood pressure, mmHg | 73.1±11.4 |
| Non-smoker | 72.0% | <80mmHg | 71.3% |
| Current smoker | 6.2% | ≥80mmHg | 28.7% |
| Ex-smoker | 21.7% | LDL-C, mmol/L | 3.1±0.9 |
| Drinking status |  | <2.6mmol/L | 30.2% |
| Non-drinker | 75.0% | ≥2.6mmol/L | 69.8% |
| Current drinker | 3.3% | Triglyceride, mmol/L | 1.7±1.1 |
| Social drinker | 10.9% | <1.7mmol/L | 63.6% |
| Ex-drinker | 10.8% | ≥1.7mmol/L | 36.4% |
| Education level |  | Total cholesterol, mmol/L | 5.0±1.1 |
| No formal education/ primary | 28.3% | <4.5mmol/L | 31.9% |
| Primary | 36.8% | ≥4.5mmol/L | 68.1% |
| Secondary/ tertiary | 30.0% | HDL-C, mmol/L | 1.2±0.3 |
| Tertiary | 4.9% | ≤1.0mmol/L male; ≤1.3mmol/L female | 54.6% |
| Duration of DM, year | 8.4±6.8 | >1.0mmol/L male; >1.3mmol/L female | 45.4% |
| ≤5 years | 34.3% | TC/HDL-C ratio | 4.5±1.5 |
| 5-10 years | 29.4% | <4.5 | 55.1% |
| >10 years | 36.3% | ≥4.5 | 44.9% |
| **clinical variables (mean±SD)** |  | Chronic kidney disease stage |  |
| BMI, kg/m^2^ | 25.1±4.1 | Stage 1 (≥90ml/min/1.73m^2^ ) | 26.0% |
| <23kg/m^2^ | 31.1% | Stage 2 (60-89ml/min/1.73m^2^ ) | 42.8% |
| ≥23kg/m^2^ & <27.5kg/m^2^ | 44.4% | Stage 3 (30-59ml/min/1.73m^2^ ) | 25.1% |
| ≥27.5kg/m^2^ & <30kg/m^2^ | 13.1% | Stage 4 (15-29ml/min/1.73m^2^ ) | 4.2% |
| ≥30kg/m^2^ | 11.4% | Stage 5 (<15ml/min/1.73m^2^ ) | 1.9% |
| Waist hip ratio | 0.9±0.1 | Urine ACR, mg/mmol | 14.6±52.8 |
| ≤0.9 male; ≤0.85 female | 20.6% | ≤ 2.5 mg/mmol man; ≤ 3.5 mg/mmol female | 61.0% |
| >0.9 male; >0.85 female | 79.4% | > 2.5 mg/mmol man; > 3.5 mg/mmol female | 39.0% |

DM=Diabetes Mellitus; BMI = Body Mass Index; HbA1c = Haemoglobin A1c; SBP = Systolic Blood Pressure; DBP = Diastolic Blood Pressure; LDL-C = Low-density Lipoprotein-Cholesterol; TC = Total Cholesterol; HDL-C = High-density Lipoprotein-Cholesterol; ACR = Albumin/Creatinine ratio;
